# Optimized Biodiesel Production from Waste Cooking Oil (WCO) using Calcium Oxide (CaO) Nano-catalyst

**DOI:** 10.1038/s41598-019-55403-4

**Published:** 2019-12-12

**Authors:** Tadesse Anbessie Degfie, Tadios Tesfaye Mamo, Yedilfana Setarge Mekonnen

**Affiliations:** 1 Center for Environmental Science, College of Natural and Computational Sciences, Addis Ababa University, P. O. Box 1176, Addis Ababa, Ethiopia; 2 Ministry of Innovation and Technology (Ethiopia), P. O. Box 2490, Addis Ababa, Ethiopia; 3 Ministry of Mine and Petroleum (Ethiopia), P. O. Box 486, Addis Ababa, Ethiopia

**Keywords:** Environmental impact, Environmental chemistry

## Abstract

Biodiesel production from waste cooking oil (WCO) provides an alternative energy means of producing liquid fuels from biomass for various uses. Biodiesel production by recycling WCO and methanol in the presence of calcium oxide (CaO) nano-catalyst offers several benefits such as economic, environmental and waste management. A nano-catalyst of CaO was synthesized by thermal-decomposition method and calcinated at 500 °C followed by characterization using x-ray diffraction (XRD) and scanning electron microscope (SEM) techniques. The XRD results revealed nano-scale crystal sizes at high purity, with a mean particle size of ~29 nm. The SEM images exhibited morphology of irregular shapes and porous structure of the synthesized nanocatalysts. The highest conversion of WCO to biodiesel was estimated to be 96%, at optimized experimental conditions i.e., 50 °C, 1:8 WCO oil to methanol ratio, 1% by weight of catalyst loading rate and 90 minutes reaction time, which is among few highest conversions reported so far. Biodiesel properties were tested according to the American (ASTM D6571) fuel standards. All reactions are carried-out under atmospheric pressure and 1500 rpm of agitation.

## Introduction

Accessibility of energy sources and climate change are the two biggest challenges that mankind facing in this century. The fast-growing population and the increasing prosperity have led to rapid rise in the energy demand. Human civilization predominantly depends on the utilization of energy, it plays a big role in socio-economic development by improving the standard of living. Energy is vital for the economic development of every country. Every sector of the economy such as agriculture, industry, transport, commercial and domestic sectors require energy^1–5^. Fossil fuel-based fuel sources such as petroleum, coal and natural gas have been the predominant sources of energy all over the world for a long time. The majority of the world energy needs, about 81.1% is supplied through petrochemical sources such as coal, oil and natural gas. Nuclear, hydro, biofuel and other renewable energy systems account only 18.9%^6^.

The high energy demand in the industrialized world as well as in the domestic sector had caused environmental pollution problems due to their widespread use of fossil fuels. Fossil fuel combustion has several public health risks and environmental problems which extend to universal and potentially irreversible consequences on global warming^1,7^. As a result, the concerns about environmental impacts have increased and trigged the examination of alternative energy sources. Typical forms of renewable energy include wind power, hydropower, solar energy, biomass and biofuels. The contribution of all these resources is important because of the economic and environmental reasons, and biodiesel could be one of the solutions^8^.

Biodiesel is a substitute to diesel fuel derived from the triglycerides of vegetable oils or animal fats. Biodiesel can be produce from various vegetable oils such as palm oil, sunflower, soybean, rapeseed and castor oil using different types of catalysts^1,4,5,9–11^. In this article, the phrase “waste cooking oil” refers to edible oil which has formerly been used for frying in restaurants and hotels, and no longer be used for similar purpose. In most towns in developing countries including in Ethiopia waste cooking oil simply dumping into the environment. This causes serious environmental, social, economic and health problems to the society^11–15^. Improper or poor waste cooking oil disposal into water bodies rises the level of organic pollutants in the water. This significantly lowers the water quality and consequently affects the life of fish stocks, other aquatic living things and the surrounding community^11^.

Biodiesel production from WCO is environmentally friendly for it recycles waste cooking oil and gives renewable energy with lower pollution. It substitutes some amount of petrochemical oil import and also lowers the cost of waste management. Biodiesel production from waste cooking oil has three solutions those are economic, environmental and waste management^7,8,16,17^. Heterogeneous catalysts have attracted great attention in recent times for use in the biodiesel production^18^. The need for development of heterogeneous catalysts has risen due to the fact that homogeneous catalysts used for biodiesel production pose some limitations. These drawbacks include; washing of products with water to remove catalyst from the products which results in waste water generation and loss of biodiesel as a result of washing, the use of intensive biodiesel separation protocol, the corrosive nature of the catalysts and impossibility of catalyst reuse. Heterogeneous catalysts have also the advantages of easy separation from the product, reusability and eco-friendly. Calcium oxide nanoparticle has a higher basicity, non-corrosive, can be synthesized with a lower price, lower solubility and easier to handle than homogenous catalysts. In addition to these advantages, its being safe to the ecosystem made it an interesting choice for a catalyst^4,5,8,10,11,19–23^.

The main purpose of this research work is to enhance the production of biodiesel from waste cooking oil feed stock using nanoparticles of CaO and optimizing the major transesterification reaction parameters. In this study, nano sized calcium oxide (CaO) catalyst is synthesized with high purity using thermal decomposition method and characterized using XRD and SEM techniques. The biodiesel production reaction parameters such as WCO to methanol ratio, catalyst dose and reaction temperature were optimized for optimum biodiesel yield at laboratory scale.

## Methodology

### Waste cooking oil sample preparation

Waste cooking palm oil was collected from café, restaurants and street fast food sellers in Addis Ababa city which has been used for food frying. The waste cooking oil was settled for 4−6 days at room temperature and pressure and later filtered by sieves of hole size 100 nm to remove any suspended food particles and inorganic residues and followed by heating at 110 °C for water removal.

### Nano-catalyst synthesis

CaO nano-catalyst was prepared by thermal decomposition method following the procedure of Zhen-Xing Tang and David Claveau^24^. A nitrate solution was prepared by mixing 11.81 g of calcium nitrate tetrahydrate (Ca (NO_3_)_2_.4H_2_O) was dissolved in 25 ml of ethylene glycol solution and 2.10 g of sodium hydroxide was added into above mixture under vigorous stirring. In order to get uniform size nanoparticles, after it has been stirred for 10 min, the gel solution was kept about 5 hours at static state. Then it was washed using distilled water followed by vacuum drying. Finally, different sizes of CaO nano-particles were obtained after calcination at 500 °C.

### Catalyst characterization

The synthesized catalyst properties were characterized by X-ray diffraction (XRD) for identification of major components and for the determination of crystallite size. XRD analysis was performed with Mini Flex 600 × -ray diffraction (XRD) system with Ni filtered CuKα radiation at λ = 0.154 nm and Scanning electron microscope (SEM) JSM-IT300 LV was used to study the morphology of the synthesized catalyst.

### Transesterification process

Biodiesel is produced from triglycerides in the presence of alcohol with catalyst through transesterification reaction.

The biodiesel production from waste cooking oil with methanol in the presence of nano-sized calcium oxide nano-catalyst was done at a laboratory scale. Transesterification reaction is carried out in a flask with overall volume of 300 ml flask was placed on a hot plate equipped with a controlled magnetic stirrer and temperature sensor. Waste cooking oil was preheated to the required reaction temperature before methanol and the catalyst were added into the reaction flask. The calculated amount of methanol to oil ratio was poured into the reactor. Then the CaO catalyst was added in a range between 0.5 to 5% by weight with respect to mass of the WCO, and then the formed reaction mixture was mixed for 10 minutes. 100 ml of waste cooking oil was added and temperature of the mixture was set from 30 to 70 °C, 5 °C interval. Transesterification proceeded under continuous stirring of the reaction mixture for a desired duration.

All transesterification reactions were carried-out at atmospheric pressure with stirring speed of 1500 rpm. Thermometer was inserted into the flask to monitor the reaction temperature. After the completion of the reaction, the mixture was transferred into a separating funnel and allowed to stand overnight. Three phases were formed due to the solid catalyst and glycerol is denser than biodiesel.

### Biodiesel characterization

The separated biodiesel was heated above the boiling point of methanol (64.7 °C) to remove excess unreacted methanol. Moreover, very few suspended solid catalysts are removed by settling it for two to three days then the Biodiesel viscosity, specific gravity, water and sediment, total acidity, ash content, sulfur content, Flash Point and Cloud Point were checked according to the American Society for Testing and Materials (ASTM D 6751).

## Results and Discussion

### WCO characterization

The frying process changes the chemical and physical characteristics of the oil because in the frying process many chemical reactions were carried like hydrolysis, polymerization, oxidation and material transfer between oil and food. The collected WCO sample physic-chemical properties are reported in the Table 1.

**Table 1:** Physico-chemical properties of the sample waste cooking oil.

Physicochemical Properties	Values
Physical state at 40 °C	Liquid
Color	Dark Oily
Density at 40 °C (in Kg/m^3^)	920
Ash content	0.021
Acid value (mg KOH/gm)	3.08
FFA content (Wt % of oil)	1.54
Iodine value (g I_2_/100 g)	84
Dynamic Viscosity at 40 °C in mpa.sec	58.31
Kinematic Viscosity at 40 °C in mm^2^/s	63.38

### Catalyst characterization

#### XRD analysis

**Figure 1:**
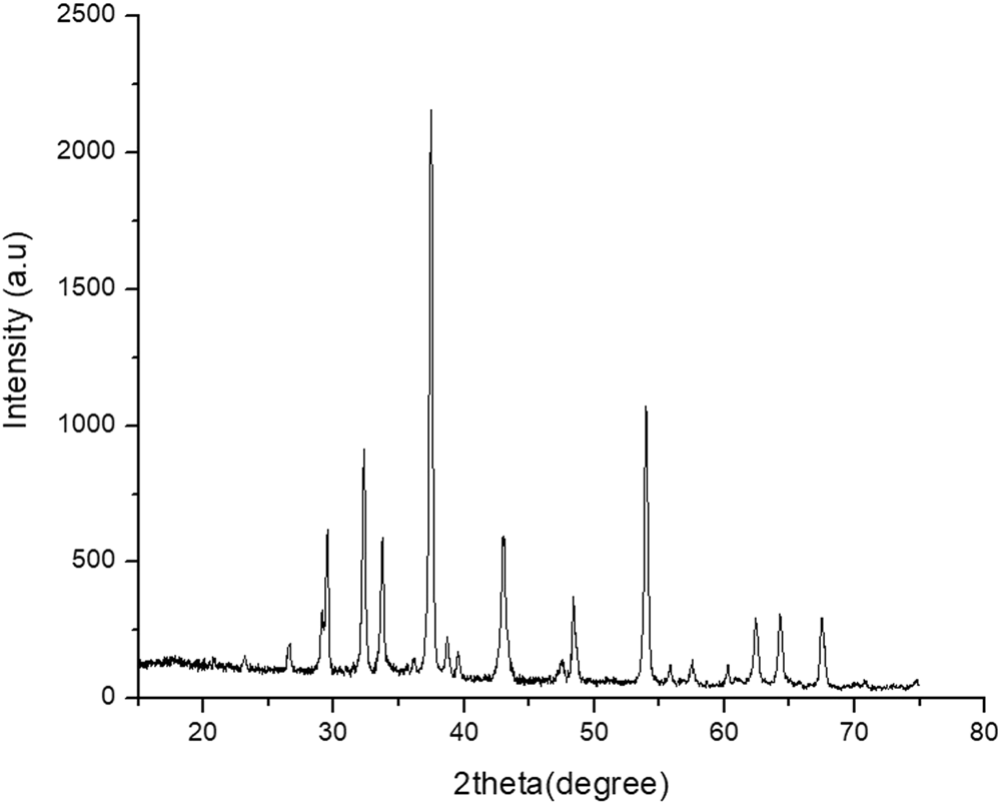
XRD result of the synthesized CaO nano-catalyst.

As the XRD diffraction intensity (pattern) of CaO nanoparticle were present in Fig. 1 and the 2θ value of the synthesized CaO Catalyst was seen in the range 15–70°.

As can be seen in Fig. 1, the sharp spectra revealed high crystallinity of the powder. The sharp peaks were exhibited at 2-theta (2θ) of 32.25°, 37.41°, 43.03°, 53.92° and 64.2°. The crystallite size diameter (D) in nanometer of the CaO nanoparticle were calculated by using Debye Scherrer equation (D = Kλ/β cos θ) and as it was seen in Table 2 the particle size of the synthesized CaO lies between 27.02 nm and 31.21 nm, with the mean crystal size of 29.072 nm.

**Table 2:** Results of XRD pattern at the peak values.

Two theta value in Degree	K-constant	β-beta FWMH	λ - X-ray wavelength in nm	Intensity	D-(size) In nm
32.25	0.94	0.32°	0.1540598	384.3	27.0
37.41	0.94	0.32°	0.1540598	1000	27.37
53.92	0.94	0.32°	0.1540598	496.8	29.14
64.2	0.94	0.32°	0.1540598	123.7	30.62
67.39	0.94	0.32°	0.1540598	95.6	31.21

#### SEM analysis

**Figure 2:**
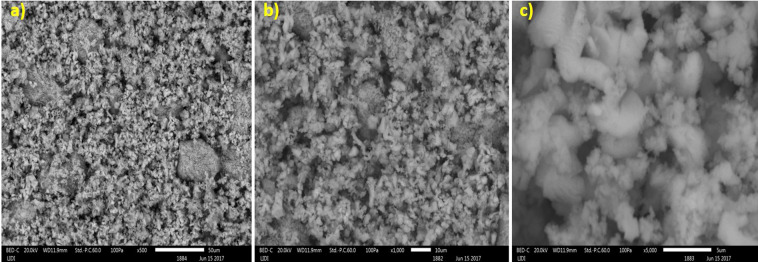
(**a**–**c**) shows the SEM images of synthesized CaO nano-catalyst at 50 μm, 10 μm and 5 μm magnification, respectively.

The Scanning Electron Microscopy (SEM) analysis was performed at 50 μm, 10 μm and 5 μm magnifications as shown in Fig. 2(a–c), respectively. According to the SEM images, the prepared CaO nano-catalyst typically comprises irregular shape of particles, porous in structure and possesses active sites. In other words, there were various sizes and shapes of particles, which indicate that the catalyst has bigger surface area for reaction.

### Optimization of reaction parameters

#### Catalysts loading

**Figure 3:**
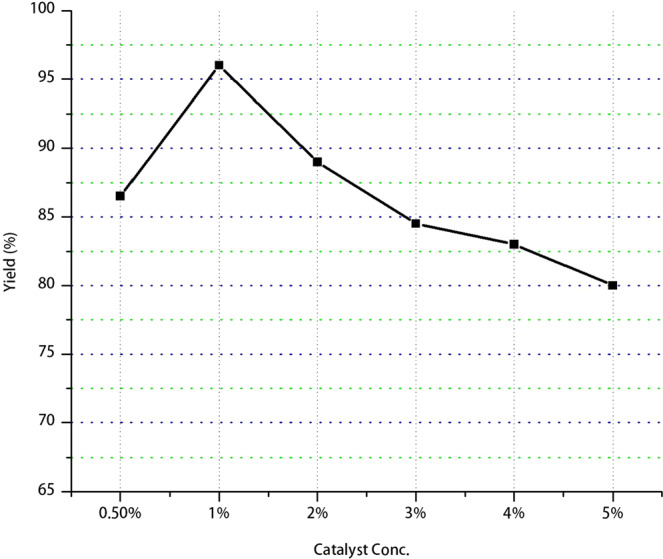
Influence of the amount of CaO Catalyst (%) on biodiesel yield (%).

To take in to consideration of the catalyst impact on the biodiesel yield amount a baseline reaction without catalyst were carried at reaction temperature at 50 °C, reaction time at 90 minutes and oil to methanol ratio at 1:8 reactions and it gives no biodiesel. Hence, presence and amount of Catalyst used plays a vital role in the optimization process of biodiesel production in the transesterification reaction. In this research work the amount of catalyst used on the yield of biodiesel amount was investigated by varying the amount of the percentage mass of the catalyst range from 0.5% to 5% w/w with the mass of WCO and keeping constant the reaction temperature at 50 °C, reaction time at 90 minutes and oil to methanol ratio at 1:8 as shown in Fig. 3.

As it can be seen from the result the biodiesel yield increases as of the catalyst concentration increases from 0.5 to 1% w/w while further increment in catalyst loading concentration shows a decrement in Biodiesel yield. Accordingly, the optimal catalyst amount was note at 1% w/w catalyst loading with 96% biodiesel yield. The excess catalyst has slightly reduced the biodiesel yield because the excess catalyst amount reaction soap formation also increase and it hinders further biodiesel production^25^. Unlike other heterogeneous nano catalysts nano-CaO were prepared without much effort and it only needs preparation and activation by calcination of the prepared catalyst^26^. In addition, it is not expensive, environmental friendly, easy to handle, low solubility in organic solvents with high basicity and reusability nature^26–28^. Reusability of CaO was not done in this research work. However, in many researches it can be seen that the Nano-CaO catalyst can be reused with no significant catalytic decrement from three^26^ to six^28^ times.

#### WCO to methanol molar ratio

The influence of the variable oil to methanol molar ratio on the yield of biodiesel was studied for the ratios 1:4, 1:5, 1:6, 1:7, 1:8, 1:9 and 1:10. The stoichiometric molar ratio of triglyceride to methanol in the transesterification reaction is 1:3. So, 1:4 was taken as the starting value for oil to methanol ratio. Oil to methanol ratio has been varied from 1:4 to 1:10 by keeping constant the reaction temperature at 50 °C, reaction time at 90 minutes and a keeping the 1% optimum value of the catalyst amount with the mass of the WCO and the percentage change in yield has been observed.

**Figure 4:**
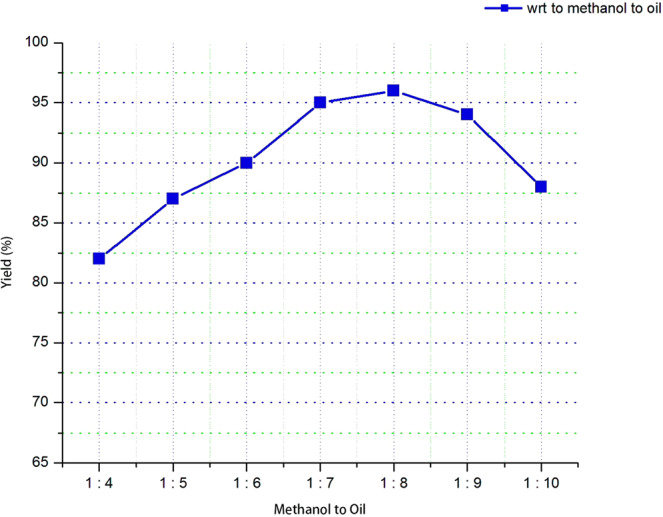
The effect of WCO to methanol ratio range from 1:4 to 1:10 on biodiesel yield (%).

As it is clearly shown in Fig. 4 that the molar ratio of oil to methanol has a substantial impact on the yield of biodiesel. When the molar ratio increases from 1:4 to 1:8 the biodiesel yields likewise increases. The optimal oil to methanol molar ratio was determined to be 1:8 with a biodiesel yield of 96%. In order to enhance the rate of methanolysis the amount of methanol must be found in excess to promote the formation of methoxy species on the surface of catalyst. This will shift the equilibrium towards the biodiesel formation. Moreover, the biodiesel yield slightly reduced when the oil to methanol molar ratio was higher than the optimal ratio, 1:8. Furthermore, the presence of excess alcohol in the product affects the quality of biodiesel fuel by reducing its viscosity, density and flash point^29^. Transesterification process yield glycerol as a by-product of the reaction. Glycerol is highly dissolve in the excessive methanol and later hinder the reaction of methanol to reactants and catalyst. Therefore, this makes the separation of glycerol from the product very challenging and allowing the equilibrium to shift in the reverse direction and consequently lowers the biodiesel yield.

#### Reaction temperature

**Figure 5:**
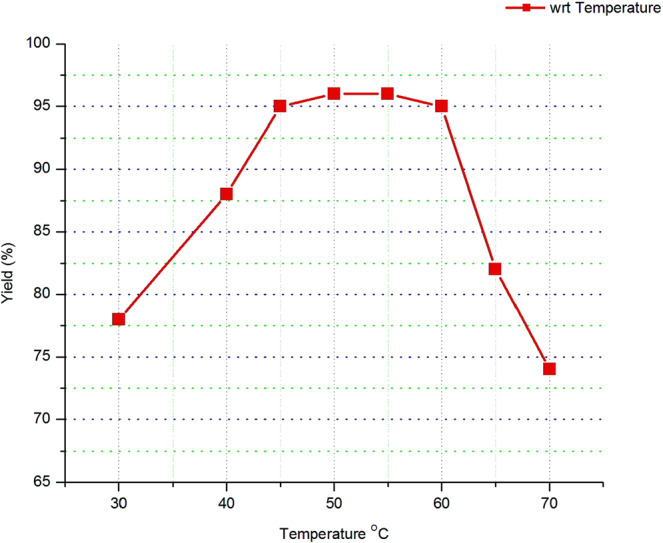
The effect of reaction temperature on biodiesel yield (%).

By keeping Methanol to oil molar ratio and catalyst loading constant on their optimum value (1:8 and 1%) and Varying the reaction temperature from 30 °C to 70 °C has given the result as shown in Fig. 5. That show that the yield of biodiesel from waste cooking oil at different reaction temperature from 30 to 70 °C.

The yield of biodiesel increases as of the temperature increases till 50 °C which is the optimal point and it gives 96% biodiesel yield. Above this temperature the yield was decreased sharply and reaches 74% yield at 70 °C. The applied thermal energy must be sufficient enough in order to overcome the diffusion resistance developed within the three phases of the reaction mixture (i.e., oil-alcohol-catalyst). However, applying temperature beyond the optimal range is not preferred. Since as the temperature reaches around the boiling point of methanol, it will rapidly vaporize and produce a large number of bubbles, which hinders the reaction and consequences the decrease in the biodiesel yield.

#### Reaction time

**Figure 6:**
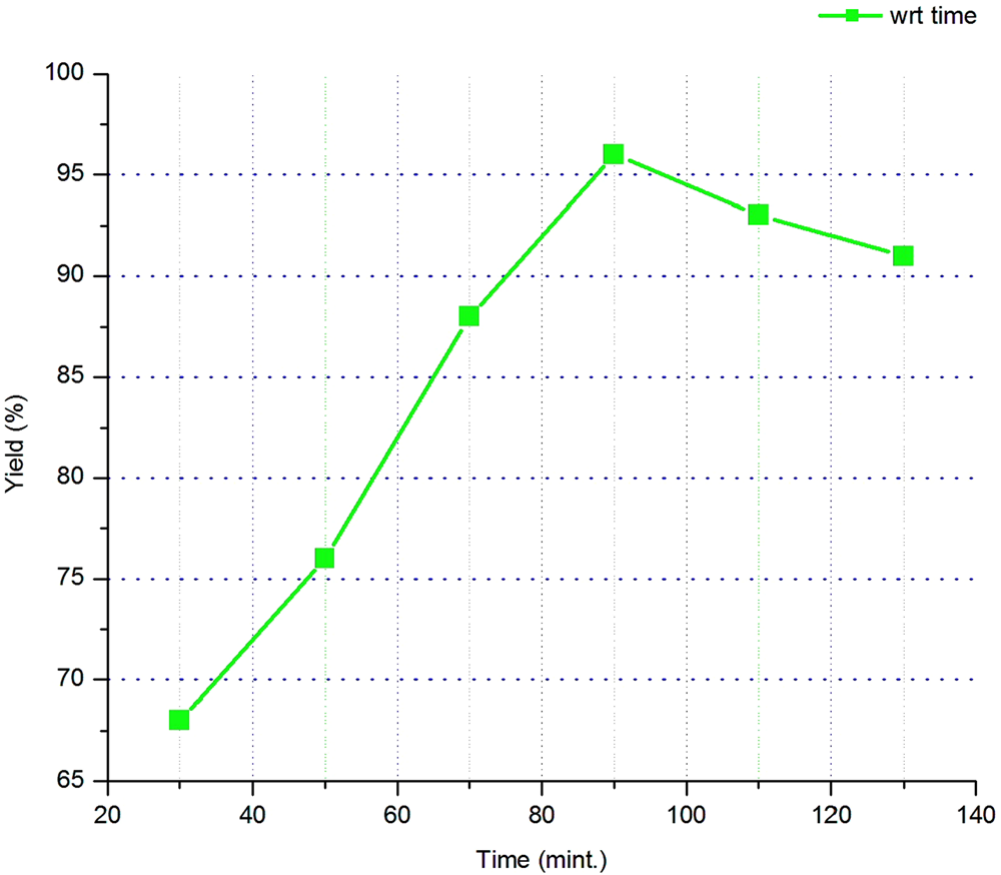
The effect of reaction time (minute) on biodiesel yield (%).

Varying the reaction time from 30 to 130 minutes and keeping the molar ratio of methanol to oil ratio, the catalysts loading amount and the temperature constant on their optimum value has given the result as shown in Fig. 6. That shows the yield of biodiesel from waste cooking oil through transesterification reaction at different reaction duration from 30 to 130 minutes.

In the first stages of the transesterification reaction the forward reaction or the production of the biodiesel was fast until it reaches equilibrium. However, the backward reaction was start after reactions carried beyond the optimal reaction duration. Hence, too long reaction duration reduces the biodiesel yield. in conjunction, determining the optimum reaction duration for transesterification is vital and in this research work case the optimum reaction duration was 90 minutes with a yield of 96% biodiesel.

### Overall effects of reaction parameters

**Figure 7:**
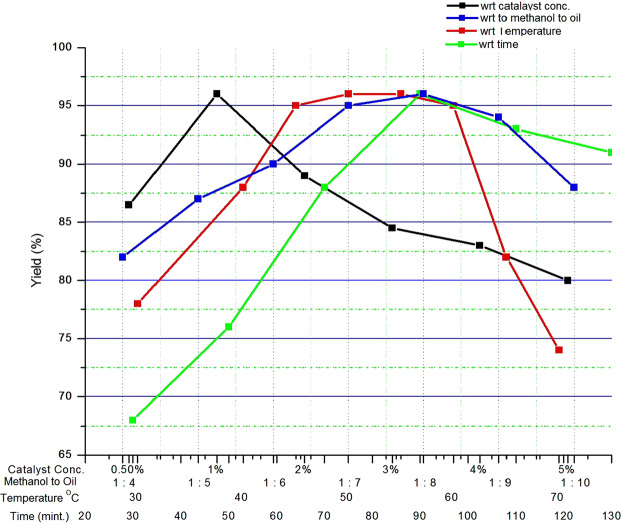
The overall effect of reaction parameters on biodiesel yield (%).

As shown in Fig. 7, the experimental results revealed a maximum yield of 96.0% (w/w), therefore it was concluded that the maximum amount of biodiesel yield was gained at 1% (w/w) of catalyst loading, 1:8 oil to methanol molar ratio at 50 °C temperature and 90 minutes of reaction duration.

The produced biodiesel viscosity, specific gravity, water and sediment, total acidity, ash content and sulfur content were check according to ASTM D6571 and it is found to be in a good agreement, see Table 3. Biodiesel from waste cooking oil could be used as a diesel fuel and for cleaner household energy source for cooking.

**Table 3:** Characteristics of produced biodiesel, compared with standard values.

Property	Test Method ASTM	EPSE Diesel Limits	ASTM D6751, limit for B100	Test Result
Density at 15 °C, g/ml	D4052	Report	—	0.8798
Density at 20 °C, g/ml	D4053	Report	—	0.8763
Flash Point (PMCC-FPT), ^o^C	D93	Min. 60	Min. 93	96
Cloud point, ^o^C	D2500	Max. + 5	Report	9
Kinematic Viscosity at 40 ^o^C mm^2^/sec	D445	1.9–6.0	1.9–6.0	5.2
Water and sediment, % v/v	D2709	Max. 0.05	Max. 0.05	0.02
Total Acidity, mg KOH/g	D974	—	Max. 0.500 (D874)	0.497
Ash content, mass %	D482	Max. 0.01	Max. 0.01 (D874)	Trace
Sulfur content, mass%	D4294	Max. 2	Max. 0.05	0.0143

## Conclusion

A CaO nano-catalyst, with a mean particle size of 29 nm, was synthesized by thermal decomposition method and used as a catalyst for biodiesel production in the transesterification process from WCO. The optimal biodiesel yield of 96% was achieved at optimized reaction conditions i.e., WCO to methanol molar ratio of 1:8, 1 wt. % of CaO nano-catalyst, 50 °C reaction temperature and 90 minutes reaction time. The produced biodiesel viscosity, specific gravity, water and sediment, total acidity, ash content and sulfur content were tested according to the American fuel standards (ASTM D 6571) and found in good agreement of the standard. Biodiesel from WCO could be used as a diesel fuel and for cleaner household energy source for cooking which was considered as renewable energy and environmental recycling process from waste vegetable oil after frying.
