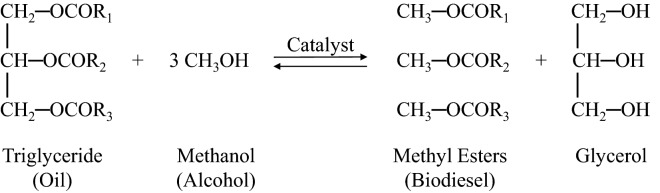# Author Correction: Optimized Biodiesel Production from Waste Cooking Oil (WCO) using Calcium Oxide (CaO) Nano-catalyst

**DOI:** 10.1038/s41598-023-32726-x

**Published:** 2023-04-11

**Authors:** Tadesse Anbessie Degfie, Tadios Tesfaye Mamo, Yedilfana Setarge Mekonnen

**Affiliations:** 1 Center for Environmental Science, College of Natural and Computational Sciences, Addis Ababa University, P. O. Box 1176, Addis Ababa, Ethiopia; 2 Ministry of Innovation and Technology (Ethiopia), P. O. Box 2490, Addis Ababa, Ethiopia; 3 Ministry of Mine and Petroleum (Ethiopia), P. O. Box 486, Addis Ababa, Ethiopia

Correction to: *Scientific Reports* 10.1038/s41598-019-55403-4, published online 12 Dec 2019

This Article contains an error in the Methodology, under the subheading ‘Transesterification process’, where the scheme incorrectly illustrates the transesterification process due to an incorrect double bond structure for the Triglyceride (Oil) and Methyl Ester (Biodiesel) parts.

The correct scheme appears below: